# Supplementary material for: BugSigDB captures patterns of differential abundance across a broad range of host-associated microbial signatures
Source: Nat Biotechnol. 2023 Sep 11;42(5):790–802. doi: 10.1038/s41587-023-01872-y (PMC11098749; doi:10.1038/s41587-023-01872-y)
Supplement: Supplementary file 1 — Supplementary Results, Figs. 1–11 and Tables 1–6. [file 41587_2023_1872_MOESM1_ESM.pdf]

nature biotechnology

Article https://doi.org/10.1038/s41587-023-01872-y

BugSigDB captures patterns of differential abundance across a broad range of host-associated microbial signatures

In the format provided by the authors and unedited

Contents

S1 Supplementary Results **2**
S1.1 Curated metadata in BugSigDB 2
S1.2 Enrichment analysis of individual CRC studies 2

List of Figures

S1 Publication date of curated papers 4
S2 Distribution of taxonomic levels 4
S3 BugSigDB Semantic MediaWiki web interface 5
S4 Comparison of semantic similarity and Jaccard similarity 6
S5 Signature similarity: antibiotics treatment 7
S6 Signature similarity: HIV infection 8
S7 Signature similarity: COVID-19 9
S8 Signature similarity: Gastric cancer 10
S9 Relationship between sample size and ranking of spike-in signatures 11
S10 Clustering of consensus signatures for body site 12
S11 Genera with mutual exclusive abundance changes between conditions 13

List of Tables

S1 Body areas and anatomical sites 14
S2 Reported measures of alpha diversity 16
S3 Body sites with frequently reported changes in alpha diversity 16
S4 Conditions with frequently reported changes in alpha diversity 16
S5 Individual CRC studies from curatedMetagenomicData in BugSigDB 16
S6 ORA of CRC studies from curatedMetagenomicData in BugSigDB 17

S1 Supplementary Results

S1.1 Curated metadata in BugSigDB reveals common practices in human microbiome research

BugSigDB provides curated metadata that enable stratification of microbiome signatures by study design, sample size, and evidence type (Table 1 of the main manuscript). Recorded lab analysis fields include sequencing type (16S: 92.5%; metagenomic shotgun (MGX): 7.5%) and sequencing platform (Illumina: 68%; Roche 454: 15%; Ion Torrent: 7.2%; RT-qPCR: 6.6%). Most of the 16S studies amplified the V4 region, which has implications on which taxonomic clades can be reliably detected [1, 2].
A side benefit of the database is a survey of the popularity of different statistical tests in the published literature. Non-parametric tests for testing for differences in mean microbial abundance between two sample groups (Mann-Whitney U test, 29.2%) or more than two groups (Kruskal-Wallis rank test, 8.1%) were most frequently used, often performed using the popular `LEfSe` tool (28.4%) for metagenomic biomarker discovery [3]. Considerable fractions also employed parametric tests based on the raw read counts (via `DESeq2` [4], 7.3%) or relative abundance (using a t-test, 6.3%) for differential abundance testing. Recently suggested tools for differential abundance tests accounting for the compositionality of microbiome data [5] were rarely used.
As differential abundance of individual microbes can also be a side effect of systematic differences in alpha diversity between the contrasted sample groups, BugSigDB records whether and which measures of alpha diversity were reported. For most experiments, alpha diversity was either unchanged (410, 33.4%) or not reported (399, 32.6%), and roughly equal numbers of experiments reported either increased (187, 15.3%) or decreased (229, 18.7%) alpha diversity in the study group. Most frequently reported measures of alpha diversity were Shannon diversity (reflecting number of species and their relative abundance, 38.3%) and richness (number of species, 22.4%, Supplementary Table S2).

S1.2 Enrichment analysis of individual CRC studies from curatedMetagenomicData in BugSigDB

The individual CRC studies from `curatedMetagenomicData` (cMD) were not included in BugSigDB at the time of writing the manuscript and creating Figure 3 of the main manuscript. For reproducibility, all analyses presented in the manuscript have been carried out based on the BugSigDB v1.0.2 release (Jan 25, 2022). Figure 3A of the main manuscript reports the results of an over-representation analysis of BugSigDB signatures in the set of differentially abundant genera obtained from comparing fecal metagenomes of pooled cohorts of 662 CRC patients and 653 healthy controls from 10 cMD datasets. The two meta-analytic signatures, which are themselves derived from large pooled cohorts and are expected to report robust CRC vs. healthy abundance changes, have thus been included as spike-in / positive control signatures. The individual cMD studies that have small sample sizes are anticipated to also report spurious signatures. Given the relationship between sample size and ranking of the spike-in signatures shown in Figure 3C of the main manuscript, one would not necessarily expect all signatures derived from the individual datasets to be strongly enriched, and some might simply not report a sufficient number of differentially abundant taxa to be included in the enrichment analysis.
During the review phase of this manuscript, we confirmed this by repeating the analysis with a more recent snapshot of BugSigDB (`b87f34e`, Jan 29, 2023) which added signatures from 4 of the individual CRC studies in `curatedMetagenomicData` (Supplementary Table S5). Two of these studies (ZellerG_2014 and VogtmannE_2016) reported signatures that were too small to be included in the over-representation analysis which required a minimum of 5 genera in a signature. The other two studies comprised a medium-sized dataset (FengQ_2015, 41 CRC vs 55 healthy samples) and a large dataset (YachidaS_2019, 258 CRC vs 251 healthy samples). The resulting ranking of the signatures of FengQ_2015 and YachidaS_2019 in an

over-representation analysis of 776 BugSigDB signatures is shown in Supplementary Table S6.
In agreement with the anticipated effect of sample size, the signature from YachidaS_2019 was strongly enriched and near the top of the ranking (10 differentially abundant genera out of 13 genera total in the signature, Benjamini-Hochberg adjusted p-value $2.5 \cdot 10^{-5}$), whereas the signature from FengQ_2015 did not show a strong enrichment (4 differentially abundant genera out of 10 genera total in the signature, Benjamini-Hochberg adjusted p-value 0.16).

References

[1] Schloss, P.D.: The effects of alignment quality, distance calculation method, sequence filtering, and region on the analysis of 16S rRNA gene-based studies. PLoS Comput Biol **6**, 1000844 (2010)

[2] Yang, B., Wang, Y., Qian, P.Y.: Sensitivity and correlation of hypervariable regions in 16S rRNA genes in phylogenetic analysis. BMC Bioinformatics **17**, 135 (2016)

[3] Segata, N., *et al.*: Metagenomic biomarker discovery and explanation. Genome Biol **12**, 60 (2011)

[4] Love, M.I., Huber, W., Anders, S.: Moderated estimation of fold change and dispersion for RNA-seq data with DESeq2. Genome Biol **15**, 550 (2014)

[5] Gloor, G.B., Macklaim, J.M., Pawlowsky-Glahn, V., Egozcue, J.J.: Microbiome datasets are compositional: and this is not optional. Front Microbiol **8**, 2224 (2017)

[6] Resnik, P.: Semantic similarity in a taxonomy: an information-based measure and its application to problems of ambiguity in natural language. J Artif Intell Res **11**, 95–130 (1999)

[7] Webber, W., Moffat, A., Zobel, J.: A similarity measure for indefinite rankings. ACM Trans Inf Syst Secur **28**, 1–38 (2010)

[8] Mungall, C., *et al.*: Uberon, an integrative multi-species anatomy ontology. Genome Biol **13**, 5 (2012)

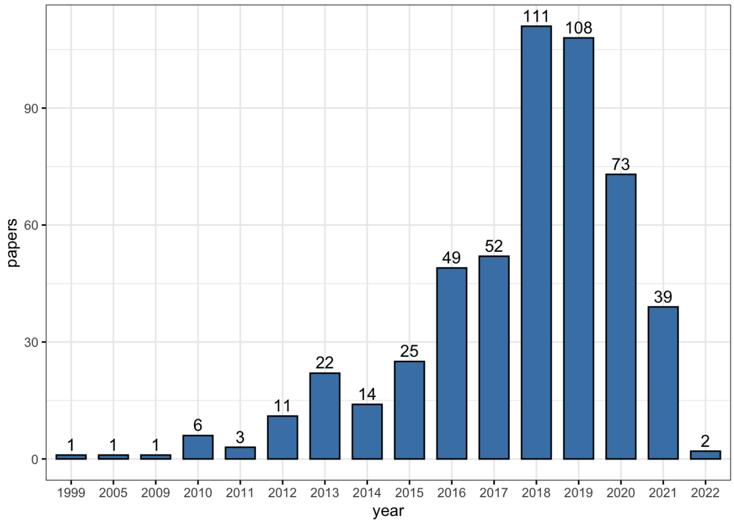

Figure S1: Publication date of curated papers. The curated papers cover two decades of human microbiome research, with the majority of studies being published in the last 5 years (385 / 526 studies, 73.2%).

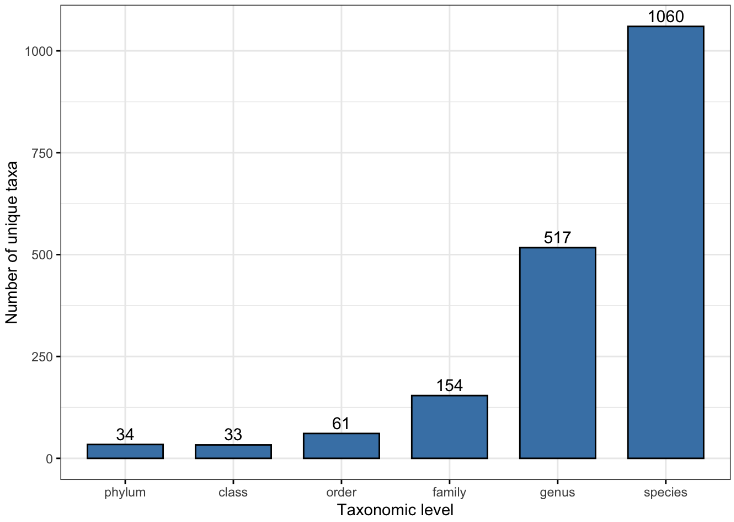

Figure S2: Distribution of taxonomic levels in BugSigDB signatures. Shown is the number of unique taxa (y-axis) for each taxonomic level on the x-axis.

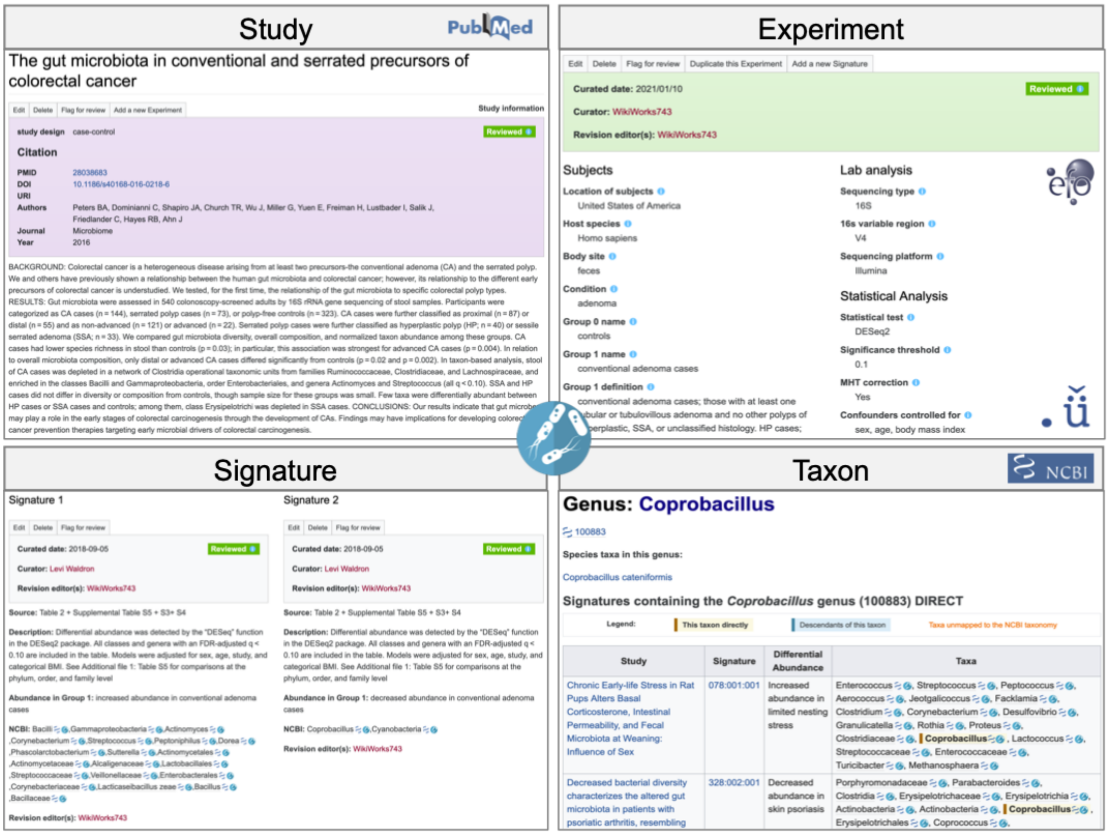

Figure S3: A semantic MediaWiki for microbial annotations. Semantic MediaWiki web interface available at https://bugsigdb.org for data entry, semantic validation, and web-based programmatic access to annotations for individual microbes and microbe signatures. This includes dedicated pages for (a) studies annotated with study design and linked to PubMed, (b) experiments describing characteristics of enrolled study participants, experimental procedures, and statistical analysis based on established ontologies and controlled vocabulary for body site and disease condition, (c) signatures specifying curation source and direction of abundance change (increased / decreased) of (d) individual taxa annotated following the nomenclature of the NCBI Taxonomy Database, facilitating also direct comparison of entered signatures to existing signatures in the database.

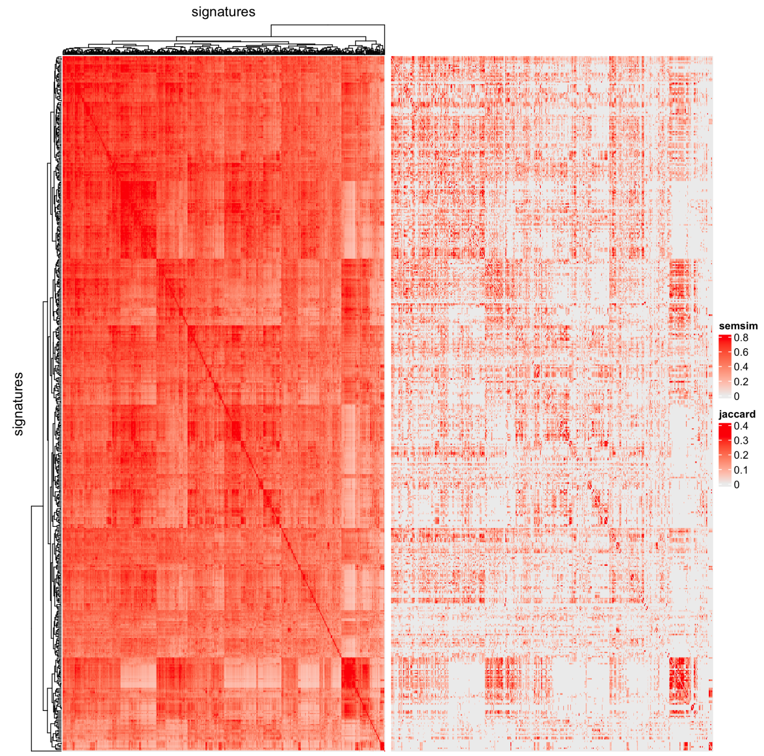

Figure S4: Comparison of semantic similarity and Jaccard similarity. We applied two different approaches for computing similarity between signatures: (1) the more restrictive Jaccard index based on pairwise overlaps between signatures harmonized to genus level (right panel), and (2) the more sensitive semantic similarity (left panel) based on taxonomic distance between signatures of mixed taxonomic levels (see Methods, main manuscript). Hierarchichal clustering of signature similarity for both similarity measures was in good agreement, but demonstrated better resolution of semantic similarity compared to the sparse results obtained from the application of Jaccard similarity.

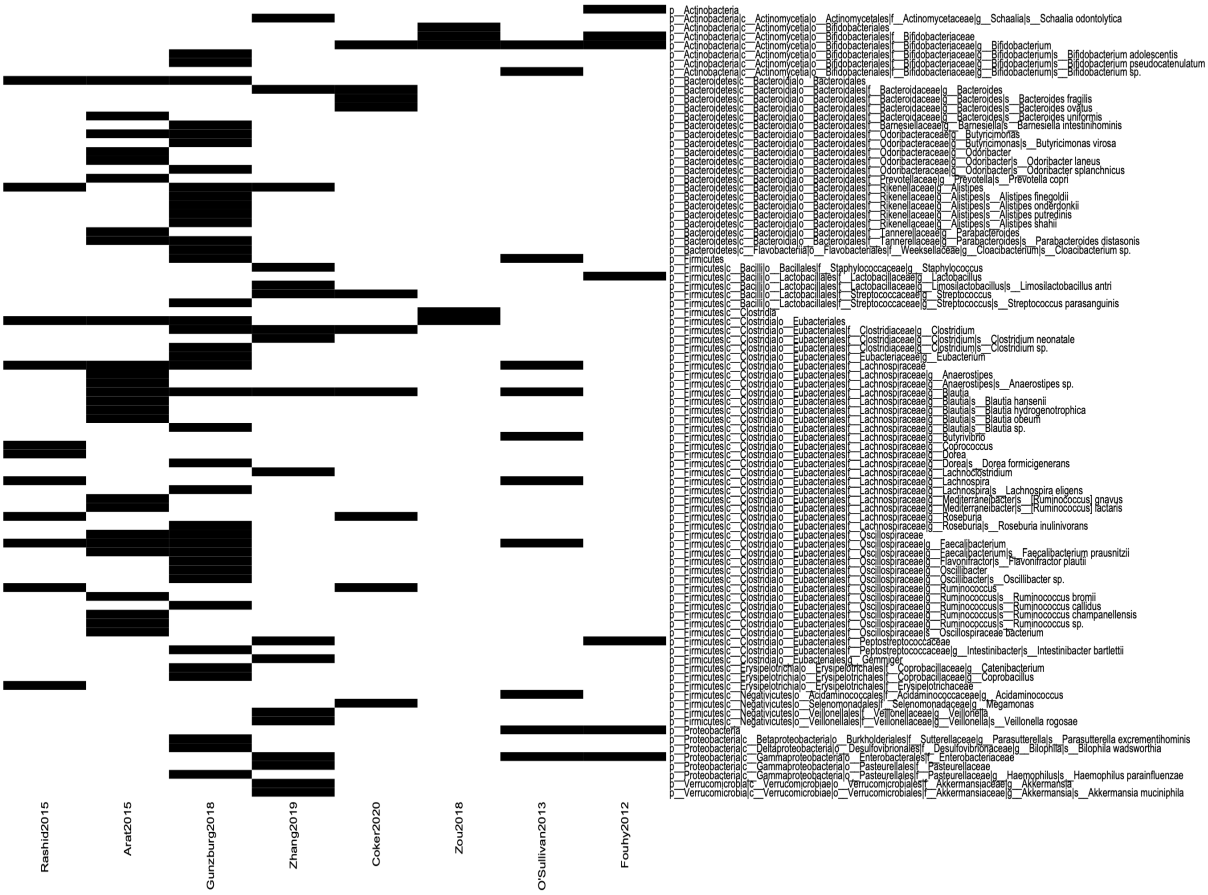

Figure S5: Similarity between fecal signatures of decreased abundance after antibiotics treatment. Microbial contents of the signatures shown in Figure 2D and E of the main manuscript (x-axis) delineating the taxa contained in these signatures (y-axis). Signatures contain mixed taxonomic levels from phylum to species, which is taken into account through the computation of semantic similarity between signatures.

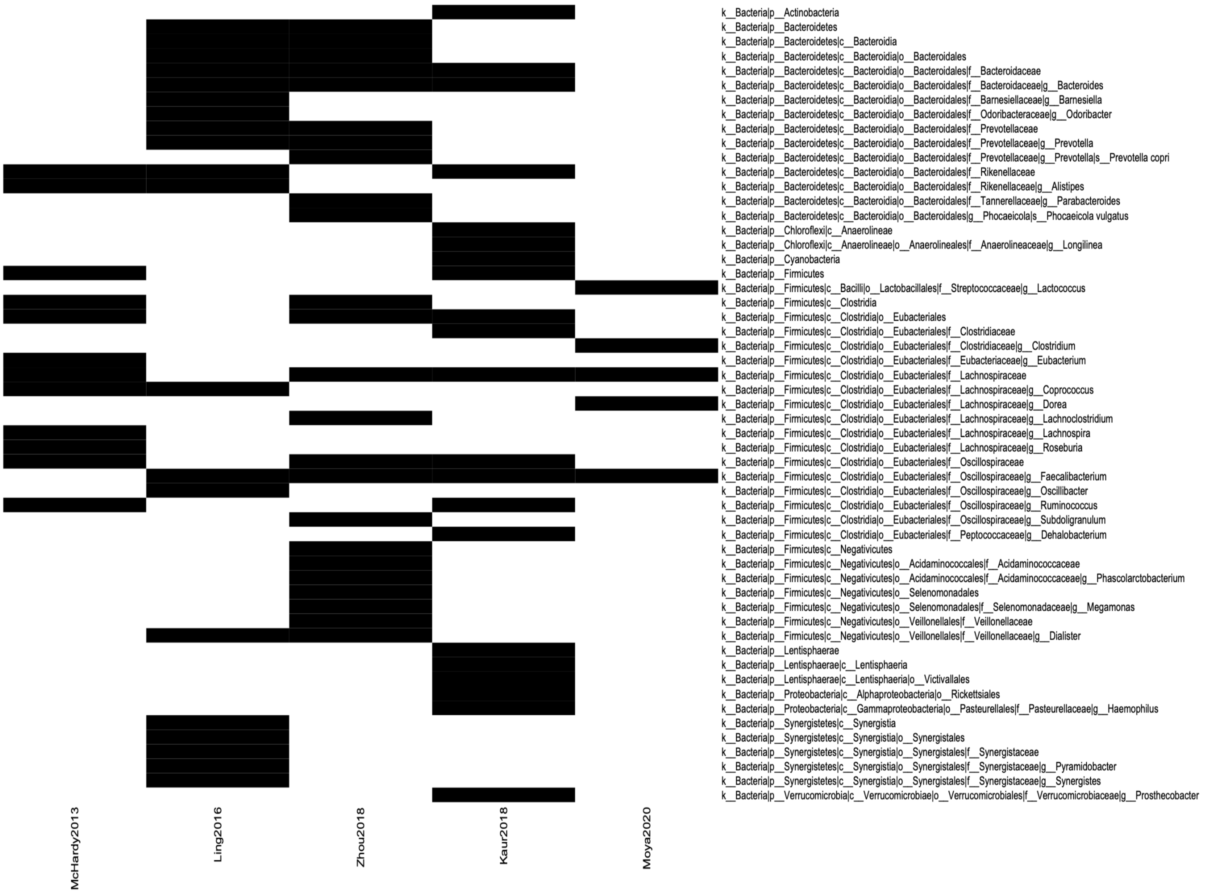

Figure S6: Similarity between fecal signatures of decreased abundance in HIV infection. Microbial contents of the signatures shown in Figure 2B and C of the main manuscript (x-axis) delineating the taxa contained in these signatures (y-axis). Signatures contain mixed taxonomic levels from phylum to species, which is taken into account through the computation of semantic similarity between signatures.

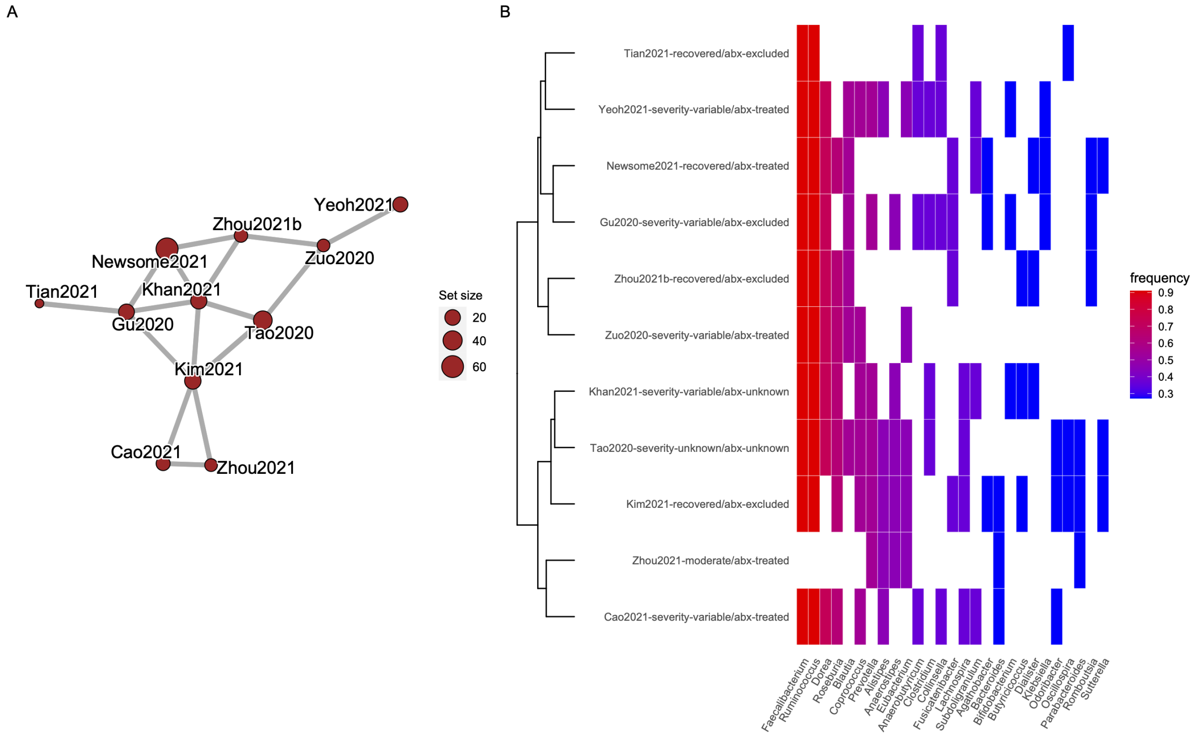

Figure S7: Similarity between fecal signatures of decreased abundance in COVID-19. (A) Semantic similarity between signatures. Each node corresponds to a signature. The size of each node is proportional to the number of taxa in a signature. More similar signatures are connected by shorter and thicker edges. **(B)** Microbial contents of the signatures (x-axis) delineating the taxa contained in these signatures (y-axis). COVID-19 severity and antibiotics (abx) treatment is indicated in the signature name.

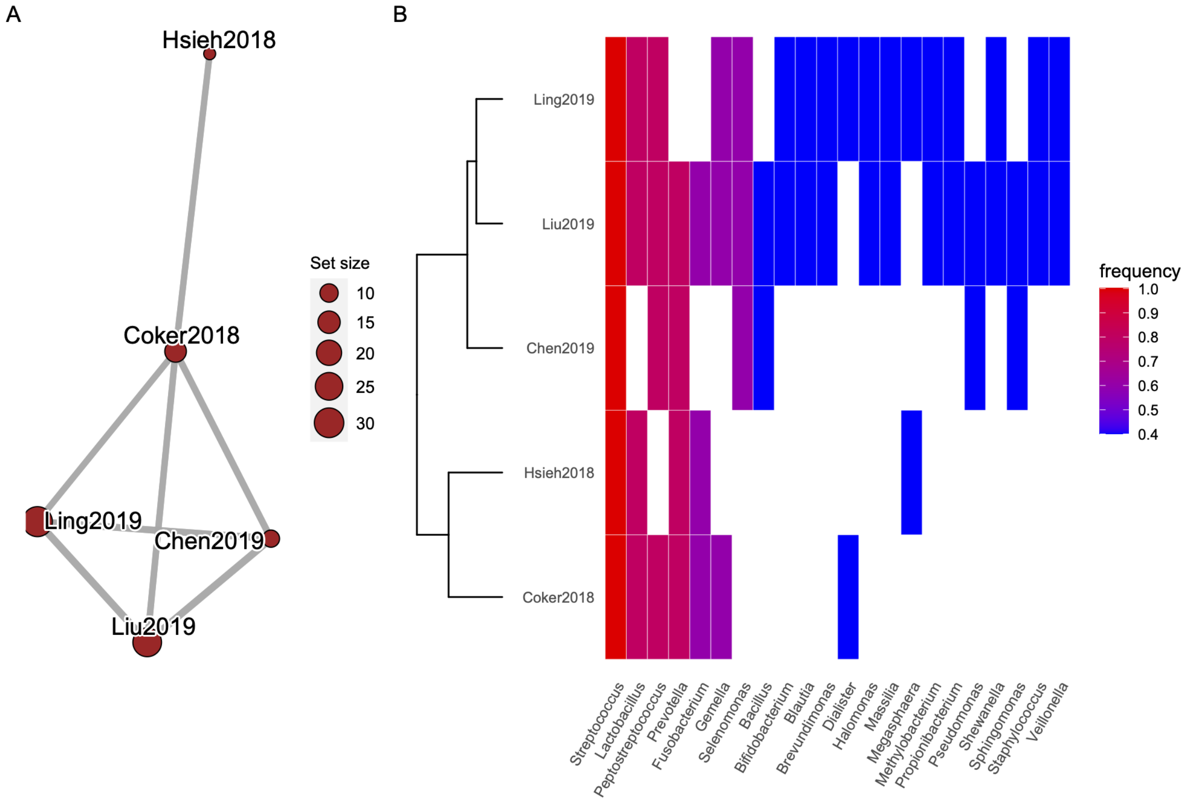

Figure S8: Similarity between stomach signatures of increased abundance in patients with gastric cancer. **(A)** Semantic similarity between signatures. Each node corresponds to a signature. The size of each node is proportional to the number of taxa in a signature. More similar signatures are connected by shorter and thicker edges. **(B)** Microbial contents of the signatures (x-axis) delineating the taxa contained in these signatures (y-axis).

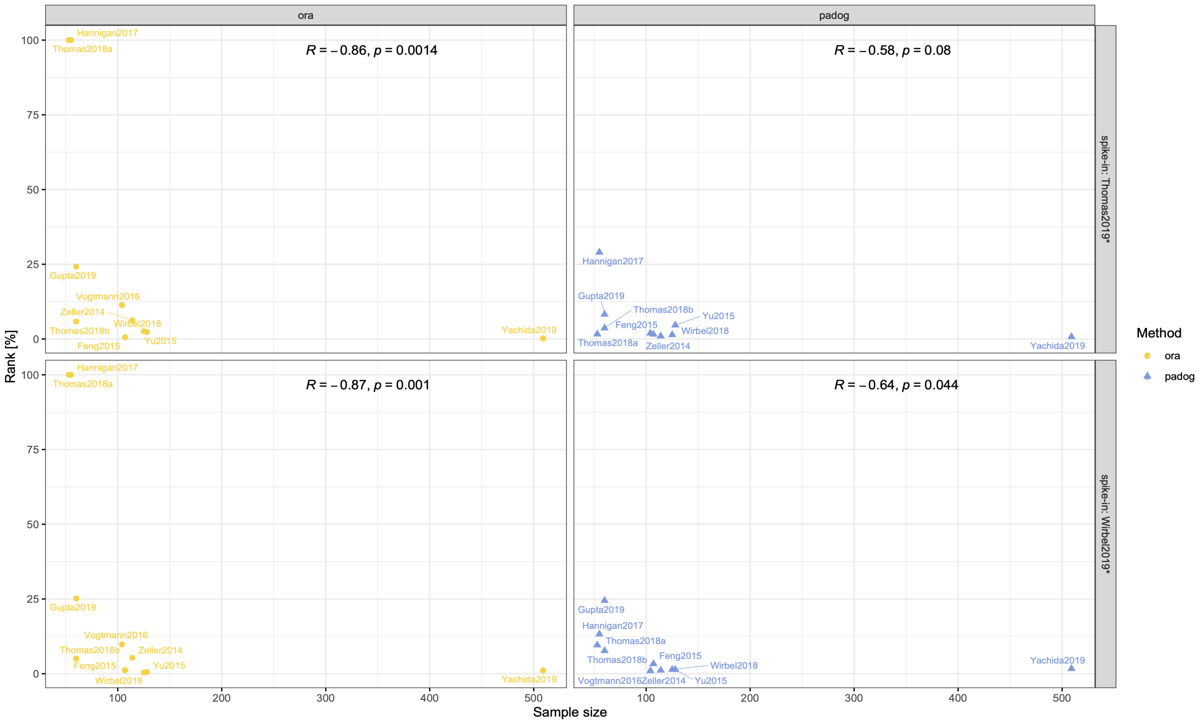

Figure S9: Relationship between sample size and ranking of spike-in signatures. Relative ranks (y-axis) of both spike-in signatures for ORA and PADOG when applied to 10 published metagenomic datasets of varying sample size (x-axis). The correlation and p-value of a two-sided Spearman's correlation test is annotated to each panel. A general trend of better ranking of the spike-in signatures for larger sample sizes is apparent for both methods, although the impact of lack in power for smaller sample sizes is stronger for ORA.

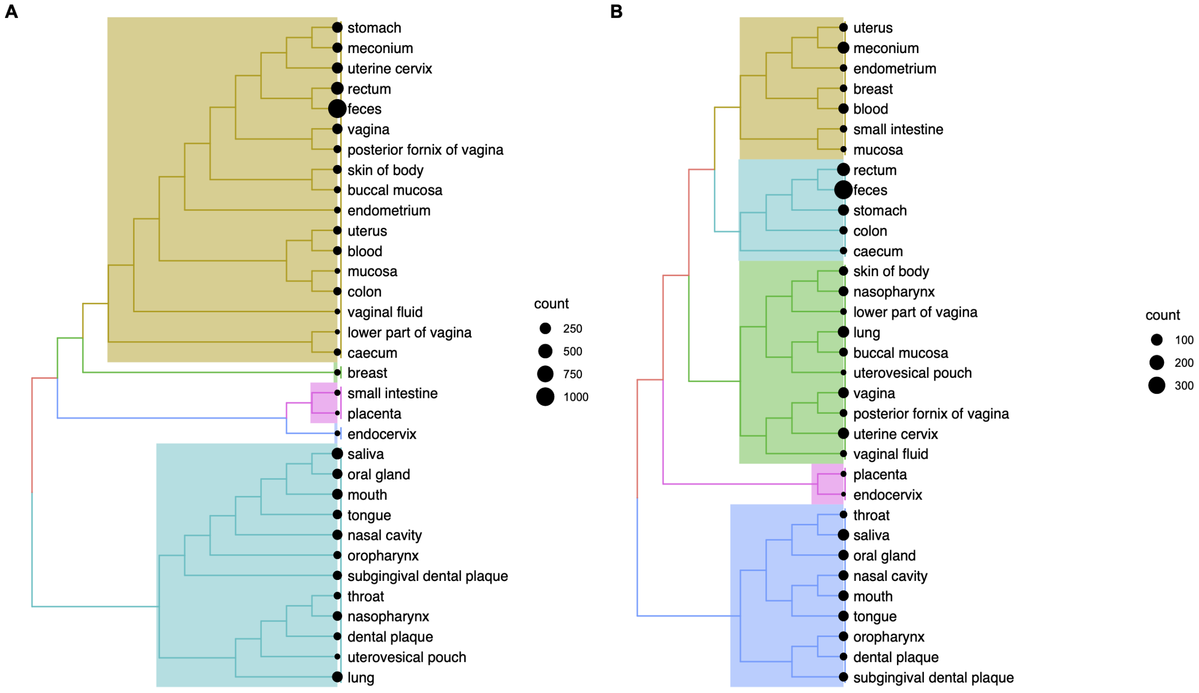

Figure S10: Clustering of consensus signatures for body site. (A) Clustering of weighted meta-signatures containing mixed taxonomic levels by semantic similarity [6], and **(B)** Clustering of weighted genus-level signatures by rank-biased overlap [7].

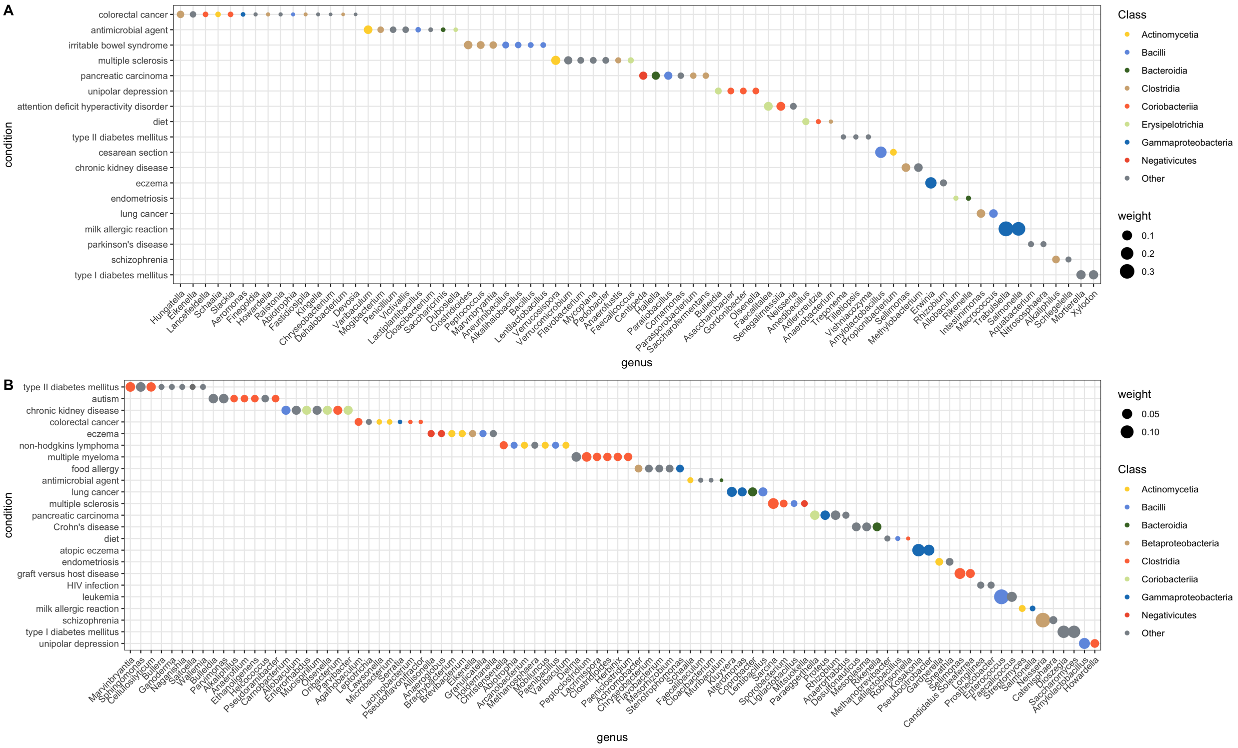

Figure S11: Genera with mutual exclusive abundance changes between conditions. Shown are the genera (x-axis) that are exclusive to one of the conditions on the y-axis, i.e. genera that are only reported for this condition with **(A)** increased or **(B)** decreased abundance in the study group. The weight / size of the dot corresponds to the relative sample size reporting this genus in a meta-signature for the corresponding condition.

Table S1: Body areas and anatomical sites. Microbiome studies in BugSigDB investigate microbiome samples from 14 broad body areas comprising more than 60 refined anatomical sites standardized based on the UBERON Anatomy Ontology [8].

Body area	Anatomical site	UBERON ID
Oral	Mouth	UBERON:0000165
	Lower lip	UBERON:0001835
	Buccal mucosa	UBERON:0006956
	Oral cavity	UBERON:0000167
	Oral opening	UBERON:0000166
	Oropharynx	UBERON:0001729
	Pharyngeal mucosa	UBERON:0000355
	Saliva	UBERON:0001836
	Tongue	UBERON:0001723
	Gingiva	UBERON:0001828
	Internal cheek pouch	UBERON:0013640
	Dental plaque	UBERON:0016482
	Subgingival dental plaque	UBERON:0016484
	Throat	UBERON:0000341
	Hypopharynx	UBERON:0001051
Nasal	Nose	UBERON:0000004
	Nasal cavity	UBERON:0001707
	Nasopharynx	UBERON:0001728
Respiratory tract	Lung	UBERON:0002048
	Bronchus	UBERON:0002185
	Sputum	UBERON:0007311
Upper GI tract	Stomach	UBERON:0000945
	Mucosa of stomach	UBERON:0001199
	Duodenum	UBERON:0002114
	Duodenal mucosa	UBERON:0000320
Lower GI tract	Colon	UBERON:0001155
	Colonic mucosa	UBERON:0000317
	Intestine	UBERON:0000160
	Intestinal mucosa	UBERON:0001242
	Large intestine	UBERON:0000059
	Caecum	UBERON:0001153
	Small intestine	UBERON:0002108
	Mucosa of small intestine	UBERON:0001988
	Ileum	UBERON:0002116
	Rectum	UBERON:0001052
	Mucosa of rectum	UBERON:0003346
	Feces	UBERON:0001988
	Meconium	UBERON:0007109
Skin	Skin of body	UBERON:0002097
	Skin of cheek	UBERON:0008803
	Skin of forearm	UBERON:0003403
	Skin of penis	UBERON:0001331
	Skin of sole of pes	UBERON:0013778
	Interdigital space	UBERON:0036252

Vaginal	Vagina	UBERON:0000996
	Lower part of vagina	UBERON:0015243
	Vaginal fluid	UBERON:0036243
	Posterior fornix of vagina	UBERON:0016486
Female reproductive system	Uterus	UBERON:0000995
	Uterine cervix	UBERON:0000002
	Uterovesical pouch	UBERON:0011049
	Endocervix	UBERON:0000458
	Endometrium	UBERON:0001295
	Ovary	UBERON:0000992
	Placenta	UBERON:0001987
Male reproductive system	Prostate gland secretion	UBERON:0004796
	Semen	UBERON:0001968
Blood	Blood	UBERON:0000178
Breast milk	Milk	UBERON:0001913
Urine	Urine	UBERON:0001088
Lymph node	Mesenteric lymph node	UBERON:0002509
Other	Breast tissue	UBERON:0000310
	Peritoneal fluid	UBERON:0001268

Table S2: Reported measures of alpha diversity. Shown are the number of experiments that reported one of the indicated alpha diversity measures in the columns with either decreased, increased, or unchanged alpha diversity in the exposed group when compared to the unexposed group.

	Shannon	Richness	Chao1	Simpson	Inverse Simpson	Pielou	Total
Decreased	150	82	98	42	14	11	397
Increased	117	93	62	33	6	4	315
Unchanged	426	235	232	164	30	25	1122
Total	703	410	392	239	50	40	1834

Table S3: Body sites with frequently reported changes in alpha diversity. Shown are the top 5 body sites most frequently reported with increased (top) or decreased (bottom) alpha diversity in the exposed sample group when compared to the unexposed sample group.

	Increased	Decreased	Unchanged
Saliva	16	6	18
Mouth	11	2	16
Posterior fornix of vagina	8	0	3
Uterine cervix	9	1	30
Vagina	9	3	24
Feces	72	132	485
Stomach	3	15	4
Skin of body	2	7	14
Caecum	1	4	2
Rectum	0	2	11

Table S4: Conditions with frequently reported changes in alpha diversity. Shown are the top 5 conditions most frequently reported with increased (top) or decreased (bottom) alpha diversity in the exposed sample group when compared to the unexposed sample group.

	Increased	Decreased	Unchanged
Air pollution	14	5	9
Human papilloma virus infection	8	1	33
Cervical cancer	5	0	5
Hypertension	4	0	2
Periodontitis	4	0	5
COVID-19	7	28	38
Antimicrobial agent	5	20	39
Gastric cancer	2	15	16
Chronic kidney disease	0	5	2
Graft versus host disease	2	7	4

Table S5: Individual CRC studies from `curatedMetagenomicData` (cMD) for which signatures of differential abundant taxa are included in BugSigDB.

cMD dataset	PMID	Study (BugSigDB)	Comments
ZellerG_2014	25432777	Study 595	no taxa on genus or species level
VogtmannE_2016	27171425	Study 612	one taxon on genus or species level
YachidaS_2019	31171880	Study 630	258 CRC vs 251 healthy samples
FengQ_2015	25758642	Study 631	41 CRC vs 55 healthy samples

Table S6: Ranking of the signatures of FengQ_2015 and YachidaS_2019 in an over-representation analysis of 776 BugSigDB signatures.

cMD dataset	samples	genera	DA genera	FDR	Rank
YachidaS_2019	509	13	10	$2.5 \cdot 10^{-5}$	13
FengQ_2015	96	10	4	0.16	281
