# Supplementary material for: BugSigDB captures patterns of differential abundance across a broad range of host-associated microbial signatures
Source: Nat Biotechnol. 2023 Sep 11;42(5):790–802. doi: 10.1038/s41587-023-01872-y (PMC11098749; doi:10.1038/s41587-023-01872-y)
Supplement: Supplementary file 2 — Reporting Summary [file 41587_2023_1872_MOESM2_ESM.pdf]

nature portfolio

Corresponding author(s): Levi Waldron

Last updated by author(s): Jun 14, 2023

Reporting Summary

Nature Portfolio wishes to improve the reproducibility of the work that we publish. This form provides structure for consistency and transparency in reporting. For further information on Nature Portfolio policies, see our Editorial Policies and the Editorial Policy Checklist.

Statistics

For all statistical analyses, confirm that the following items are present in the figure legend, table legend, main text, or Methods section.

n/a	Confirmed	
☐	☒	The exact sample size (*n*) for each experimental group/condition, given as a discrete number and unit of measurement
☐	☒	A statement on whether measurements were taken from distinct samples or whether the same sample was measured repeatedly
☐	☒	The statistical test(s) used AND whether they are one- or two-sided<br>*Only common tests should be described solely by name; describe more complex techniques in the Methods section.*
☐	☒	A description of all covariates tested
☐	☒	A description of any assumptions or corrections, such as tests of normality and adjustment for multiple comparisons
☐	☒	A full description of the statistical parameters including central tendency (e.g. means) or other basic estimates (e.g. regression coefficient) AND variation (e.g. standard deviation) or associated estimates of uncertainty (e.g. confidence intervals)
☐	☒	For null hypothesis testing, the test statistic (e.g. F, t, r) with confidence intervals, effect sizes, degrees of freedom and P value noted<br>*Give P values as exact values whenever suitable.*
☒	☐	For Bayesian analysis, information on the choice of priors and Markov chain Monte Carlo settings
☒	☐	For hierarchical and complex designs, identification of the appropriate level for tests and full reporting of outcomes
☐	☒	Estimates of effect sizes (e.g. Cohen's *d*, Pearson's *r*), indicating how they were calculated

Our web collection on statistics for biologists contains articles on many of the points above.

Software and code

Policy information about availability of computer code

Data collection	Microbial signatures were collected through the Semantic MediaWiki web interface at https://bugsigdb.org. For reproducibility, all analyses presented in the manuscript have been carried out based on the BugSigDB v1.0.2 release (Jan 25, 2022) available from Zenodo under https://doi.org/10.5281/zenodo.5904281. BugSigDB signatures from Zenodo were obtained using the bugsigdbr R/Bioconductor package (version 1.4.0). Metagenomic datasets providing species-level relative abundance for fecal microbiomes of colorectal cancer patients and healthy controls were obtained through the curatedMetagenomicData R/Bioconductor package (version 3.6.0).
Data analysis	Statistical analysis was carried out using R-4.2.1 and Bioconductor-3.16. All analysis code is provided on GitHub (https://github.com/waldronlab/BugSigDBPaper). Jaccard similarity of BugSigDB signatures was calculated using the calcJaccardSimilarity function of the BugSigDBStats package (version 1.0.0). Semantic similarity was computed based on Lin's measure of semantic similarity as implemented in the ontologySimilarity R package (version 2.5). For genus-level bug set enrichment analysis of colorectal cancer signatures, species-level counts were summed across branches using the splitByRanks function from the mia R/Bioconductor package (version 1.6.0). Differential abundance analysis was carried out following the limma-trend approach as implemented in the limma R/Bioconductor package (version 3.54.0). Read counts were transformed to log counts-per-million (CPMs) using the cpm function of the edgeR R/Bioconductor package (version 3.40.0). ORA and PADOG were carried out as implemented in the EnrichmentBrowser R/Bioconductor package (version 2.28.0). CBEA was carried out as implemented in the CBEA R/Bioconductor package (version 1.2.0). For the analysis of pooled signatures, signatures were pooled for one body site at a time, and within body sites for one condition at a time, as implemented in the getMetaSignatures function of the bugsigdbr package (version 1.4.0). Semantic similarity of weighted meta-signatures was calculated by incorporating the weights into the best-match average combination approach as implemented in the weightedBMA function of the BugSigDBStats R package (version 1.0.0).

For manuscripts utilizing custom algorithms or software that are central to the research but not yet described in published literature, software must be made available to editors and reviewers. We strongly encourage code deposition in a community repository (e.g. GitHub). See the Nature Portfolio guidelines for submitting code & software for further information.

Data

Policy information about availability of data

All manuscripts must include a data availability statement. This statement should provide the following information, where applicable:

- Accession codes, unique identifiers, or web links for publicly available datasets
- A description of any restrictions on data availability
- For clinical datasets or third party data, please ensure that the statement adheres to our policy

BugSigDB is available via a Semantic MediaWiki web interface at https://bugsigdb.org, under open-source and open-data licenses described at https://bugsigdb.org/Project:About. Weekly and semi-annual snapshots are provided in plain text file formats at https://github.com/waldronlab/BugSigDBExports for cross-language and cross-application compatibility; unprocessed snapshots are available as csv files at https://bugsigdb.org/Help:Export. The companion bugsigdbr R/Bioconductor package provides advanced data manipulation, including ontology-aware and taxonomy-aware features (https://bioconductor.org/packages/bugsigdbr). The NCBI Taxonomy database is available at https://www.ncbi.nlm.nih.gov/taxonomy. The Experimental Factor Ontology is available at https://www.ebi.ac.uk/efo. The UBERON Anatomy Ontology is available at https://www.ebi.ac.uk/ols/ontologies/uberon.

Human research participants

Policy information about studies involving human research participants and Sex and Gender in Research.

Reporting on sex and gender	Not applicable
Population characteristics	Not applicable
Recruitment	Not applicable
Ethics oversight	Not applicable

Note that full information on the approval of the study protocol must also be provided in the manuscript.

Field-specific reporting

Please select the one below that is the best fit for your research. If you are not sure, read the appropriate sections before making your selection.

☒ Life sciences ☐ Behavioural & social sciences ☐ Ecological, evolutionary & environmental sciences

For a reference copy of the document with all sections, see nature.com/documents/nr-reporting-summary-flat.pdf

Life sciences study design

All studies must disclose on these points even when the disclosure is negative.

Sample size	Sample size of both contrasted sample groups is recorded for all studies curated on bugsigdb.org. The median total sample size across all 628 studies in the BugSigDB 1.0.2 release is n=25. For the bug set enrichment analysis of colorectal cancer signatures, differential abundance was calculated between n=662 colorectal cancer samples and n=653 control samples from 10 datasets. For the taxon co-occurence analysis, prevalence of frequently reported genera was calculated in n=9,623 stool samples from healthy adult controls of 68 different studies.
Data exclusions	For the bug set enrichment analysis of colorectal cancer signatures, only signatures containing 5 or more genera associated with any condition were included. Analysis of pooled signatures was restricted to body sites and conditions studied by at least two studies in BugSigDB and containing at least 5 taxa in the resulting pooled signature. Antibiotics exclusion criteria, if provided by the authors, are also recorded for studies curated on bugsigdb.org (median exclusion time across studies = 60 days).
Replication	To assess replication by independent studies of the same condition, we compared semantic similarity between signatures reported for a single condition to the similarity of randomly sampled signatures in repeated simulation, and ranked conditions based on the resulting empirical p-value. Antibiotics treatment and HIV infection were the experimental conditions with the highest level of consistency in signatures reported by independent studies. Among the enriched signatures of the bug set enrichment analysis of colorectal cancer signatures, the signatures from Wu et al., 2013 and Allali et al., 2018, are notable because neither study was included in the 10 datasets from which the meta-analysis signatures from Thomas et al., 2019 and Wirbel et al., 2019 were computed, therefore providing independent replication of these signatures.
Randomization	BugSigDB signatures are available primarily from observational study designs: case-control (281 studies, 44.7%) and cross-sectional studies (171 studies, 27.2%) were most prevalent, while prospective cohort studies (66, 10.5%), time-series/longitudinal studies (51, 8.1%), laboratory studies (29, 4.6%), randomized controlled trials (28, 4.4%), and meta-analyses (6, 0.9%) are also present. For analyses presented in this manuscript randomization does not apply, as this is not a randomized control trial.
Blinding	BugSigDB signatures are available primarily from observational study designs: case-control (281 studies, 44.7%) and cross-sectional studies (171 studies, 27.2%) were most prevalent, while prospective cohort studies (66, 10.5%), time-series/longitudinal studies (51, 8.1%), laboratory

studies (29, 4.6%), randomized controlled trials (28, 4.4%), and meta-analyses (6, 0.9%) are also present. For analyses presented in this manuscript blinding does not apply, as this is not a randomized control trial.

Reporting for specific materials, systems and methods

We require information from authors about some types of materials, experimental systems and methods used in many studies. Here, indicate whether each material, system or method listed is relevant to your study. If you are not sure if a list item applies to your research, read the appropriate section before selecting a response.

Materials & experimental systems

n/a	Involved in the study
☒	☐ Antibodies
☒	☐ Eukaryotic cell lines
☒	☐ Palaeontology and archaeology
☒	☐ Animals and other organisms
☒	☐ Clinical data
☒	☐ Dual use research of concern

Methods

n/a	Involved in the study
☒	☐ ChIP-seq
☒	☐ Flow cytometry
☒	☐ MRI-based neuroimaging
